# CONSECUTIVE POOR SLEEP AND ITS ASSOCIATIONS WITH DAILY RUMINATION AND MEMORY LAPSES

**DOI:** 10.1093/geroni/igad104.1923

**Published:** 2023-12-21

**Authors:** Soomi Lee, Kyoungmin Cho

**Affiliations:** The Pennsylvania State University, University Park, Pennsylvania, United States; Korea Advanced Institute of Science and Technology, Daejeon, Republic of Korea

## Abstract

Emerging research has shown that consecutive sleep loss impairs daily affective and physical well-being. Yet, little is known about the effects of consecutive poor sleep (in both quantity and quality) on daily cognitive stress. This study examined whether and how consecutive nights of poor sleep quantity and quality are associated with day-to-day trajectories of rumination and memory lapses. Participants were adults (n=938) from the Midlife in the United States Study III who provided data for eight diary days. Consecutive poor sleep quantity and quality were operationalized as the within-person numbers of consecutive nights with < 6 hours of sleep and with reports of very bad/bad sleep quality, respectively. Multilevel models evaluated the linear, quadratic, and cubic effects of consecutive poor sleep on daily rumination and memory lapses, after controlling for sociodemographic, health, daily covariates, and between-person means of sleep quantity and quality. Daily rumination increased in curvilinear fashion such that it increased (linear), but the rate of increase decelerated (quadratic) and then accelerated again (cubic) as the number of consecutive poor sleep quantity and quality increased. Daily memory lapses increased in linear fashion as the number of consecutive poor sleep quantity (but not sleep quality) increased. Findings show that the day-to-day trajectory of rumination is characterized by high initial reactivity, followed by decrease due to habituation and adaptation, and then increase, as poor sleep is accumulated more consecutive times. The day-to-day trajectory of memory lapses is mostly characterized by a dose-response relationship with the number of consecutive poor sleep quantity.

